# GABA Metabolism and Transport: Effects on Synaptic Efficacy

**DOI:** 10.1155/2012/805830

**Published:** 2012-02-23

**Authors:** Fabian C. Roth, Andreas Draguhn

**Affiliations:** Institute of Physiology and Pathophysiology, University of Heidelberg, 69120 Heidelberg, Germany

## Abstract

GABAergic inhibition is an important regulator of excitability in neuronal networks. In addition, inhibitory synaptic signals contribute crucially to the organization of spatiotemporal patterns of network activity, especially during coherent oscillations. In order to maintain stable network states, the release of GABA by interneurons must be plastic in timing and amount. This homeostatic regulation is achieved by several pre- and postsynaptic mechanisms and is triggered by various activity-dependent local signals such as excitatory input or ambient levels of neurotransmitters. Here, we review findings on the availability of GABA for release at presynaptic terminals of interneurons. Presynaptic GABA content seems to be an important determinant of inhibitory efficacy and can be differentially regulated by changing synthesis, transport, and degradation of GABA or related molecules. We will discuss the functional impact of such regulations on neuronal network patterns and, finally, point towards pharmacological approaches targeting these processes.

## 1. Introduction

Activity within neuronal networks is contained between the extremes of complete silence and exceeding neuronal discharges. This general statement may seem intuitively right but has severe and nontrivial consequences for the function of neuronal networks. Several theoretical arguments and experimental findings support the notion that specific mechanisms secure a limited mean level of activity. Information content within neuronal networks is maximal under conditions of sparse coding, which means that only a minority of all local neurons is activated above threshold [1]. Furthermore, neurons are severely damaged by both extremes, that is, prolonged inactivity [2–5] or severe hyperactivity during epileptic seizures [6].

**Figure 1:**
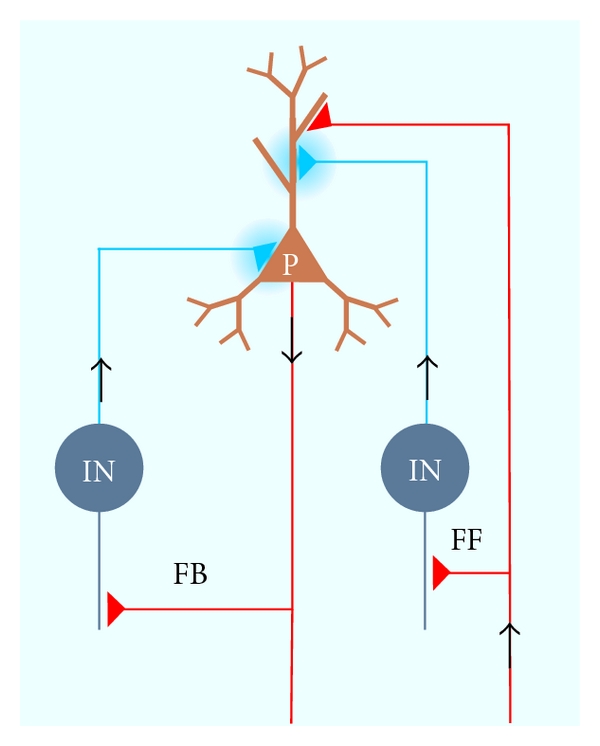
Local inhibitory connections of cortical networks. Note the efferent and afferent connections indicated by arrows. In red, connections indicate glutamatergic excitation and blue connections GABAergic inhibition. Brown soma indicates an excitatory pyramidal cell (P), and blue-grey somata show inhibitory interneurons (INs). The left interneuron is integrated into a feedback inhibition loop, (FB) while the right interneuron shows feed-forward inhibition (FF). Differential targeting by the interneurons to the soma or dendrite points towards possible layer-specific actions of inhibition. Note that GABA released at the right synapse may, eventually, spill over to the neighbouring glutamatergic synapse. The global light blue staining indicates background GABA concentration that mediates tonic inhibition depending on local synaptic activity.

Many different mechanisms contribute to regulation of overall neuronal activity, including intrinsic neuronal properties [7, 8] and energy metabolism [9, 10]. At the core of homeostasis, however, is the interplay between synaptic excitation and inhibition (Figure 1). All neuronal circuits of higher animals contain excitatory and inhibitory transmitter systems forming intense feed-forward and feedback connections [11, 12]. The functional architecture of such networks can already explain homeostatic regulation of activity to a certain degree, and excitatory feedback loops tend to build up activity, which is counterbalanced by dampening actions of inhibitory feedback connections. A further element of cortical and subcortical microcircuits is inhibition of inhibitory neurons, resulting in a net excitation of downstream target cells. This mechanism may serve further functions in synchronizing neuronal activity and can be mediated by specialized interneurons [13]. In contrast, interactions between inhibitory neurons may also desynchronize neurons as, for example, Renshaw cells in the spinal cord [14, 15]. This mechanism may serve to reduce fatigue of muscle fibers.

It should be noted that inhibitory neurons are not only important for balancing excitation. In several circuits, inhibitory neurons function as projection cells, rather than interneurons. For example, major projections within the basal ganglia and reticular nucleus of the thalamus and of the cerebellum are formed by GABAergic neurons [16–18]. In such networks, activity-dependent modulation of inhibition may have specific effects beyond balancing excitation, for example, the generation of specific physiological or pathological oscillation pattern [19].

Homeostasis between excitation and inhibition cannot be reduced to a simple rule of network wiring. Recent evidence shows that inhibition has multiple specific functions within neuronal networks, far beyond a simple “break” [20, 21]. Moreover, inhibitory strength is not constant but must adapt to dynamically changing patterns and degrees of network activity. It does therefore not come as a surprise that recent work has elucidated multiple mechanisms of plasticity at inhibitory synapses [4, 22–24]. An important subset of these mechanisms mediates homeostatic plasticity, that is, adaptation of inhibitory efficacy to the overall activity within a local network. Indeed, several lines of evidence suggest that GABAergic efficacy is upregulated in hyperactive networks [25–29] and downregulated under conditions of reduced activity [5, 30, 31].

Such homeostatic reactions can, in principle, be mediated by multiple pre- and postsynaptic mechanisms. A particularly important regulatory system, however, is the concentration of the main mammalian inhibitory transmitter GABA (*γ*-aminobutyric acid). This paper shall summarize the molecular elements and functional mechanisms involved in regulation of GABA concentration within vesicles, cells, and in the extracellular space. We will quote experimental evidence indicating that GABA is homeostatically regulated during physiological and pathological changes of network activity. Finally, we will consider how molecular determinants of GABA concentration can be targeted by drugs for pharmacological therapy of neurological or psychiatric diseases.

## 2. Organization of GABAergic Synapses

In the mammalian CNS, inhibition is mediated by the amino acids GABA and glycine. The GABAergic system has been intensely explored during recent years and will therefore be the main focus of this paper. As a starting point, we will briefly summarize the main functional and structural elements of GABAergic synapses.

GABA binds to two different types of receptors-ion channels and metabotropic receptors. GABA-gated ion channels are selectively permeable for chloride and bicarbonate and have reversal potentials close to Cl^−^ equilibrium (E_Cl_). These channels are mostly termed GABA_A_ receptors, but a molecularly and pharmacologically distinguishable subset has also been termed GABA_C_ receptors until recently, as discussed by Olsen and Seighart [32]. In most cases, the increase in chloride (and bicarbonate) conductance resulting from activation of ionotropic GABA receptors causes inhibition of the respective neuron, that is, decreased probability of action potential generation. This is easy to understand in cases where E_Cl_ is more negative than the membrane potential, such that opening of GABA_A_ receptors causes hyperpolarisation and enhances the distance between membrane potential and action potential threshold. However, inhibition can also be mediated by more complex biophysical mechanisms, for example, shunting of the local membrane resistance, which can also counteract excitatory inputs. Even depolarizing actions of GABA can, in certain cases, be inhibitory [33–35]. Conversely, excitatory actions of GABA may occur in specific situations, including early developmental stages [36–39] and maladaptive processes, for example, in chronic epilepsy [40, 41]. The occurrence of depolarizing GABA responses under physiological conditions is presently subject to some controversy [39, 42]. GABA_B_ receptors, in contrast, are members of the family of G-protein-coupled proteins [43] and react to GABA binding by dimerisation [44] and activation of downstream signal cascades. These include decreased probability of transmitter release and increase in pre- and postsynaptic K^+^ conductance [45, 46].

A complete survey of GABAergic mechanisms at the molecular, cellular, and network level is far beyond the scope of this paper. Rather, we will highlight three principles of organization of GABA-mediated inhibition that are particularly important for understanding how GABA regulates network activity. The molecular constituents involved in regulation of inhibitory strength are detailed below.

- GABA regulates excitability on different temporal and spatial scales. One important mechanism is tonic inhibition, which results from diffusely distributed GABA within the extracellular space of networks, thereby reducing excitability of all local neurons (Figure 1). Recent evidence has shown that tonic inhibition is of major importance for reducing firing probability of defined types of neurons within cortical networks [24, 47–49]. In some cells, this mechanism accounts for more than 50% of GABA-induced chloride conductance [50]. Background levels of GABA in neuronal tissue have been estimated to reach high-nanomolar to low-micromolar concentrations [51, 52]. In good accordance with this relatively low concentration, extrasynaptic GABA receptors have particularly high agonist affinity [47, 53, 54]. At the other extreme, phasic inhibition is mediated by locally and temporally restricted release of GABA from synaptic terminals. This action causes a short, exponentially rising and falling of the postsynaptic chloride conductance which can last from few to tens of milliseconds [50, 55]. Most GABAergic neurons seem to form such specific synaptic sites for phasic inhibition, but recent evidence indicates that there are also specialized interneurons which release GABA for tonic inhibition [56–60]. Tonic inhibition depends on special GABA receptors, which can be selectively modulated by drugs, for example, neurosteroids. These specific receptor isoforms may be important in the pathophysiology of depression [61] and withdrawal symptoms [62]. Such examples of receptor heterogeneity may well open new therapeutic chances.
- GABAergic interneurons are diverse. Work on different networks has revealed an unprecedented multitude of different GABAergic neurons which are classified by their somatic location, dendritic branching, axonal projection, afferent synaptic integration, intrinsic membrane properties, and expression of molecular markers, especially neuromodulatory peptides and calcium-binding proteins. Extensive classification systems have been established for different circuits, for example, for the rodent neocortex [63, 64] and the hippocampus [13, 65]. Moreover, introducing the juxtacellular recording technique has enabled recordings from individual interneurons in behaving animals and subsequent in-depth structural analysis [66]. These data have shown that different types of interneurons are specialized to organize different patterns of network activity [67].
- In accordance with the heterogeneity and functional specialization of different cell types, experiments and computer modelling have revealed important functions of “inhibitory” interneurons in networks beyond merely dampening excitation. Interneurons turned out to play a key role in organizing the spatiotemporal activity of local networks, especially during synchronous network oscillations [68–73]. Complementary neuroanatomical work has highlighted the structural basis for this function: interneurons have highly divergent axonal projections, cell type-specific afferent and efferent connectivity, and synchronizing mutual connections. All these properties favour synchronous rhythmic inhibition of large populations of principal cells [13, 69, 71, 74–76]. It should be noted that the connections between excitatory projection cells and inhibitory interneurons provide an automatic homeostatic mechanisms at the network level. Feed forward or feedback inhibition is driven by excitatory inputs or outputs, respectively, from remote or local excitatory neurons. This mechanism does automatically recruit inhibitory neurons in an activity-dependent manner and, hence, balance local activity (Figure 1).

## 3. Key Molecules for GABAergic Signalling

**Figure 2:**
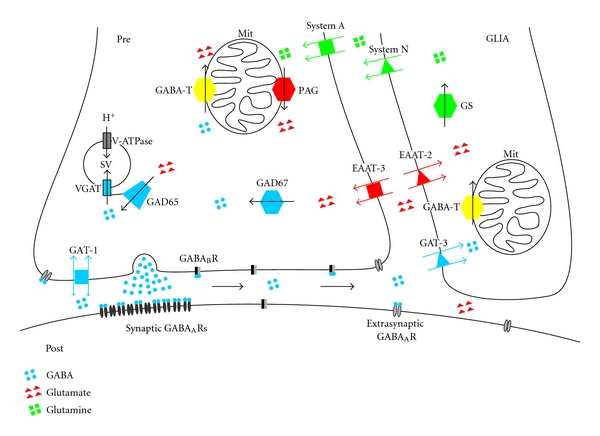
Schematic drawing of transmitter release, transport, and synthesis at a GABAergic synaptic terminal. The axonal ending of an inhibitory interneuron (PRE) is drawn on the left, a glial cell (GLIA) on the right. Bottom structure indicates postsynaptic membrane of a target cell (POST), for example, a pyramidal neuron. Transporters are marked by flanking arrows, and synthesizing or degrading enzymes are marked by a centred arrow. Transporters are colour matched to substrates: GABA is shown as blue particles, glutamate in red, and glutamine in green. GS: glutamine synthetase, Mit: mitochondrion, PAG: phosphate-activated glutaminase, SV: synaptic vesicle, and V-ATPase: vacuolar-type H^+^-ATPase. For other abbreviations, see the main text.

The molecular organization of synapses is highly complex, and a complete review would be beyond the scope of this paper. We will restrict our remarks to some families of molecules that are crucial for understanding homeostatic regulation of GABA concentration (Figure 2).

Like many other neurotransmitters, GABA acts on ionotropic as well as metabotropic ion channels. GABA_A_ receptors are pentameric ion channels composed out of a large variety of 19 homologous subunits [32, 77, 78]. Work during the past decades has elucidated numerous functional differences between molecular subtypes of GABA_A_R, including different expression patterns, differential modulation by benzodiazepines, neurosteroids and Zn^2+^, different compartmentalization within neurons, and different agonist affinity [32, 54, 79]. The latter properties are of special interest with respect to GABA concentration. GABA_A_Rs with low agonist affinity appear to be clustered at postsynaptic sites, whereas receptors with high affinity are mostly found extrasynaptically [47, 48]. The underlying sorting mechanisms are partially known and involve specific subsynaptic sorting signals within the gamma subunit and interactions with postsynaptic scaffolding proteins like gephyrin and collybistin [80–83]. Extrasynaptic receptors, in contrast, are formed by subunits mediating high agonist affinity including *α*4, *α*6, and *δ* subunits [32, 47]. This distinction reflects the different concentrations of GABA at both sites: whereas synaptically released GABA may reach transient concentrations of ~1.5–3 mM [84, 85], extrasynaptic transmitter concentration has been estimated to lie in the low micromolar range of about 0.2–2.5 *μ*M [47, 51, 52, 86]. As mentioned above, these apparently low “background” concentrations of GABA may be very efficient in regulating excitability [47–50]. An additional distinct location of GABA_A_ receptors is the presynaptic terminal itself. GABAergic auto- or heteroreceptors have been described at the axon terminals of various neurons, including spinal cord afferents [87], hippocampal mossy fibres [88], Schaffer collaterals [89], cerebellar interneurons [90], and pituitary terminals [91]. The effects of such receptors are diverse. Depending on the GABA-induced change in membrane potential and local membrane resistance, presynaptic GABA_A_ receptors may increase or decrease transmitter release [92].

GABA_B_ receptors, on the other hand, are G-protein-coupled transmembrane molecules which are activated by low concentrations of GABA and form dimers which then trigger secondary signalling cascades [43–45]. At presynaptic terminals, activation of GABA_B_Rs reduces GABA release, forming the typical negative feedback loop of autoreceptor-mediated synaptic gain control. GABA_B_ receptors are also present at glutamatergic terminals, pointing towards regular spillover of GABA from inhibitory to excitatory synapses (Figure 1, [47, 93–95]). Postsynaptically, GABA_B_Rs hyperpolarize and inhibit neurons by activating inwardly rectifying K_IR_ channels, giving rise to the “slow” or “late” phase of inhibition that follows fast, GABA_A_R-mediated effects and lasts several hundred milliseconds [96]. Furthermore, GABA_B_ receptors can also mediate tonic inhibition, exerting negative control on overall network activity [97].

## 4. GABA Transport and Synthesis

While GABA receptors act as “detectors” of local GABA concentration, the regulation of GABA itself is achieved by several specialized molecular mechanisms mediating transport, sequestration, synthesis, and the degradation of GABA. We will briefly address each class of molecules involved in these processes.

Membrane-bound GABA transporters move GABA across the cell membrane (Figure 2). The direction and efficacy of this Na^+^-coupled transport results from the driving electrochemical gradient and is directed inwardly in most situations [98, 99]. However, upon strong depolarization or altered ion homeostasis, GABA transporters can also reverse direction. This mechanism leads to nonvesicular release of GABA which may be of special importance in pathophysiological situations [60, 100, 101]. GABA transporters appear in four different isoforms with affinities around 7 *μ*M for rat GAT-1, 8 *μ*M for rat GAT-2, 12 *μ*M for rat GAT-3, and 93 *μ*M for rat BGT-1 [102–106]. Terminology of GABA transporters is not fully compatible between rats and mice [107]. In the following, we use the abbreviations for rat GABA transporters where ratGAT-1 = mouseGAT1; ratGAT-2 = mouse GAT3; ratGAT-3 = mouseGAT4; ratBGT-1 = mouse GAT2. GABA transporters are differentially expressed in the CNS. As a global rule, GAT-1 is the prevailing neuronal isoform in the rodent brain, and GAT-3 is strongly expressed in glial cells [108–110]. Expression of different GAT isoforms is, however, overlapping, so that selective modulation of one isoform will always affect more than one cell type. It might therefore turn out impossible to achieve a strictly selective block of glial or neuronal GABA uptake with conventional pharmacological tools.

An alternative pathway for enriching GABA in presynaptic terminals is transmitter synthesis from glutamate. Similar to GAT-1/3, there are membrane-bound glutamate transporter molecules at presynaptic terminals of inhibitory interneurons, namely EAAC1 (also called EAAT3) [111–113]. Moreover, neurons can synthesize glutamate from glutamine which can also be taken up by specialized transporters (see below) [114, 115]. GABAergic neurons express both mature isoforms of glutamate decarboxylase, GAD65 and GAD67 [116, 117], that convert the excitatory amino acid into GABA. The smaller isoform GAD65 is directly associated to presynaptic vesicles, indicating that glutamate, once present in the presynaptic cytosol, can be rapidly used for vesicular enrichment of GABA. Indeed, there are direct protein interactions between GAD65 and the vesicular GABA transporter VGAT (= VIAAT, vesicular inhibitory amino acid transporter), suggesting that conversion of glutamate into GABA and subsequent vesicular uptake of the transmitter may be strongly coupled processes [118].

More recently, glutamine has gained interest as an alternative source of GABA. The amino acid glutamine has long been known as the immediate precursor for glutamate. In the extracellular space, glutamine may reach concentrations of hundreds of *μ*M [119, 120]. Enrichment of glutamate in excitatory central neurons involves uptake through specific glutamate transporters by glia cells, conversion into glutamine, export via “system N” glutamine transporters, uptake into neurons by “system A” glutamine transporters, and conversion into glutamate [121, 122]. There is increasing evidence for a similar role of this glutamate/glutamine cycle in GABA synthesis. Indeed, inhibitory interneurons in the hippocampus express the system A transporter SNAT1 [115], but not SNAT2 [123]. Recordings of epileptiform activity in rodent brain slices *in vitro* have revealed functional evidence for boosting of inhibition by glutamine via this mechanism [124–127]. Using high-resolution recordings of miniature IPSCs in conjunction with pharmacological manipulation of glutamine levels and glutamine transport, these studies showed that glutamine can serve as a source for GABA, especially under conditions of increased synaptic activity. More recent evidence from rat hippocampal slices showed that the contribution of glutamine to vesicular GABA content is more pronounced in immature tissue, and that glutamine forms a constitutive source of vesicular GABA in immature hippocampal synapses on CA1 pyramidal cells. At later stages, the functional importance seems to be restricted to periods of enhanced synaptic activity [128]. This loss of function for constitutive GABA release under resting conditions goes along with an age-dependent decline in expression of SNAT1, both absolutely and in relation to the GABA-synthesizing enzyme GAD65.

## 5. Sequestration and Degradation of GABA

Within presynaptic terminals of GABAergic neurons, GABA is enriched in vesicles by the vesicular inhibitory amino acid transporter (VGAT = VIAAT). This protein is embedded in the vesicular membrane and uses the electrochemical gradient for H^+^ to shuffle GABA into small synaptic vesicles [129–133]. Additionally, chloride gradients between vesicle lumen and presynaptic cytosol may contribute to the vesicular loading of GABA [129, 131]. Interestingly, VGAT processes both major mammalian inhibitory transmitters, GABA and glycine. This is a prerequisite for the observed GABAergic/glycinergic cotransmission by single vesicles in the spinal cord [134]. Modelling studies and biochemical data suggest that vesicular GABA uptake may achieve an~1000-fold increase of the transmitter in vesicles as compared to the presynaptic cytosol [135]. On the other side, recent evidence suggests that GABAergic synaptic vesicles are leaky, implying generation of a dynamic equilibrium between accumulation and loss of GABA, given that there is enough time to reach such a steady state [132, 136]. Taking this bidirectional transport into account, the “leaky bathtub” model of synaptic vesicles comes to rather low estimates of concentration gradients between cytosol and the inner vesicle space [132, 135, 137].

Finally, GABA and *α*-ketoglutarate can be transaminated, producing succinic semialdehyde and glutamate. The reaction is catalysed by GABA transaminase (GABA-T) which is present in mitochondria of glial cells and neurons [138–140]. It is estimated that more than 90% of all GABA in the mammalian CNS is degraded in this way and contributes to energy metabolism in the tricarbonic acid cycle.

In summary, there are several different molecular pathways and compartments for enrichment, synthesis, and degradation of GABA (Figure 2). The resulting concentration of GABA in synaptic vesicles and in the extracellular space depends on the equilibrium between these mechanisms. It should be clearly stated that the absolute concentrations of GABA in the presynaptic cytosol, in vesicles, and in the extrasynaptic space are not known. The affinity constants of extrasynaptic GABA receptors may serve as a rough estimate of background concentrations (0.2–2.5 *μ*M) [86]. Direct measurements from rat cerebrospinal fluid yielded similar or slightly higher values which may be lower in humans [141].

The highly dynamic time course of transmitter concentration in the synaptic cleft, on the other hand, has been estimated based on experimental and theoretical work in different types of neurons. Peak concentrations may be as high as 0.3 to 3 mM [85, 142–147]. The cytosolic GABA concentration is most difficult to estimate or measure, especially since most of the neuronal GABA pool is used for energy metabolism rather than for synaptic inhibition.

It should be explicitly stated that none of the above-given numbers has been directly measured. Indeed, our knowledge on local GABA concentrations in different compartments is far from sufficient. This is even more concerning when we take into account the enormous heterogeneity of neurons [20, 63, 65], the different microarchitecture of different local circuits, and activity-dependent changes in GABA release and ionic homeostasis. A major challenge is the lack of quantitative data about key molecules and structures: How many GABA-uptake molecules are present at a given inhibitory synapse? What is their distribution with respect to the site of release? What is the precise extracellular volume at the synaptic cleft? How much GABA does go into glia cells and neurons, respectively? An important example for progress in this quantitative molecular approach to subcellular structure and function is the recent work on the vesicular proteasome by Takamori and colleagues [136]. 

## 6. Regulation of (GABA) in Physiology and Pathophysiology

Different lines of evidence support the view that the cellular and molecular mechanisms mentioned above make important contributions to homeostatic synaptic plasticity. This term covers changes of intrinsic and synaptic neuronal properties, which maintain the mean network activity within a determined range [4, 28]. Taking into consideration that network states change rapidly with changes in vigilance and behavioural state [148, 149], this is a nontrivial task. Individual neurons can change their activity at least by a factor of ~6 in different network patterns [150]. Nevertheless, under normal conditions, networks do neither fall into complete silence, nor into pathological hyperactivity.

Inhibition plays a critical role in network homeostasis. Most circuits contain specialized inhibitory cells which are activated by external afferent excitatory inputs (feedforward inhibition) or by collaterals from efferent excitatory axons (feedback inhibition) as illustrated in Figure 1 [11]. These inhibitory control loops ensure that excitatory neurons are inhibited in an activity-dependent manner. It should be noted, however, that inhibitory interneurons are much more than a “brake” or “gain control.” Recent evidence has revealed many other functions for these heterogeneous neurons: they are critical for organizing the complex spatial and temporal patterns of network oscillations [70, 71], selective gating of defined inputs or outputs [20], suppression of background activity [151], and precise timing of action potentials [67]. Corresponding with these specific functions, we are gaining increasing insight into the complexity of GABAergic signalling, diversity of interneurons, and plasticity of inhibitory synapses.

Notwithstanding these recent findings, however, inhibition does still have its traditional function, that is, limitation of neuronal activity. With respect to network homeostasis, this control function must adapt to changing degrees of activity in the local network. Several lines of evidence indicate that modulating GABA content of inhibitory interneurons is a key mechanism in this regulation process. For example, repetitive hyperactivity in the hippocampus of chronically epileptic rats causes upregulation of GADs, the key enzymes for production of GABA [29]. Conversely, the partial deafferentation of somatosensory cortex resulting from partial limb amputations leads to a downregulation of GABA, but not of GADs [30, 152]. These findings indicate that GABA levels are increased or decreased, respectively, in response to increasing or decreasing network activity. The underlying mechanisms are diverse with respect to time course and source of GABA.

Long-term changes in excitability, such as described above, require regulation of protein expression. Multiple studies from excitatory synapses show that changes in synaptic activity do indeed include lasting effects on protein synthesis and synaptic protein content [153, 154]. The underlying mechanisms involve calcium signalling in dendrites and nuclei [30, 155]. Much less is known about similar mechanisms in inhibitory interneurons. It would be of special importance to understand the activity-dependent regulation of key proteins such as GAD, VGAT, and others. Interestingly, BDNF (brain-derived neurotrophic factor) increases expression of GAD, indicating that neurotrophins are involved in inhibitory homeostatic plasticity. This would be well compatible with the general role of these molecules in activity-dependent plasticity [156]. Surprisingly, genes for inhibitory transmission can also be upregulated in excitatory, glutamatergic neurons following periods of enhanced activity. This intriguing finding suggests that excitatory neurons can adopt an active role in synaptic inhibition in certain situations. Such a “dual phenotype” has been clearly demonstrated in dentate granule cells, a major excitatory input cell type in the rodent hippocampus [157–159]. The axons of granule cells, called mossy fibres, form strong glutamatergic synapses on proximal dendrites of CA3 pyramidal cells and do also contact inhibitory interneurons in this region (an example of feedforward inhibition). Upon strong repetitive stimulation or following epileptic seizures, mossy fibres start expressing proteins needed for the production and vesicular storage of GABA. Electrophysiological measurements show that this GABAergic phenotype is indeed functional, giving rise to mixed excitatory and inhibitory potentials in CA3 pyramids. The GABAergic phenotype of mossy fibres seems to be more pronounced in the juvenile brain [157], consistent with the general principle of enhanced plasticity in immature neurons. While the dual phenotype of granule cells may be an extreme example, several observations indicate that similar activity-dependent changes in expression of GABAergic molecules affect the vesicular pool of GABA in typical inhibitory interneurons. For example, expression of VGAT is altered following ischemia or excitotoxic stimulation [160–162]. These changes go along with altered composition of the vesicular proteome, indicative of altered supply or release of GABAergic vesicles [163].

At a shorter time scale, GABA levels might be regulated by activity-dependent uptake of transmitter molecules. Experimental evidence for such changes came from direct injection of glutamate [164] or glutamine [124, 125] into hippocampal slices. Both approaches increased the amplitudes of miniature inhibitory postsynaptic currents (mIPSCs), indicating that the precursors had indeed been used to fuel the vesicular transmitter pool. Consistent with these findings, blocking membrane-bound transporters for glutamine, GABA, or glutamate can reduce the size of IPSCs [128, 162, 165, 166]. The relative contribution of GABA, glutamate, or glutamine uptake to the vesicular GABA pool remains, however, unknown. It can be expected that the contribution of different transmitter transporters differs among neuronal subtypes, brain regions, and developmental stages [128, 167]. However, due to the fast action of uptake molecules, it is well possible that homeostatic adaptations of intravesicular GABA concentration occur at time scales of few seconds. Strong activation of axons in the CA1 area of mouse hippocampal slices results in a rapid increase of mIPSC amplitudes, with onset time below 20 s. This increase is dependent on uptake of glutamate and GABA, indicating that increased extracellular transmitter concentrations in active neuronal networks automatically provide more “fuel” to the pool of releasable GABA, thereby constituting a negative feedback loop [165].

We have already discussed that tonic activation of GABA receptors by ambient transmitter concentrations provides a major mechanism for regulation of excitability [47, 48, 50]. It may, therefore, well be that changes in GABA uptake, production, and release cause altered tonic inhibition, possibly mediated by specialized subtypes of interneurons [56]. Quantitative knowledge about the contribution of these mechanisms is still lacking. It is also unclear how much nonvesicular release of GABA by reverse transport contributes to ambient GABA concentration. Situations of hyperactivity may favour such release mechanisms by sustained depolarization and altered local ion homeostasis [59, 60, 100, 168].

## 7. Pharmacological Use

Enhancing GABAergic inhibition is useful for the treatment of several pathological situations, including chronic pain, sleep disorders, anxiety, and—most importantly—epilepsy. In accordance with the principles outlined above, several drugs have been developed which alter presynaptic GABA content. One approach is blocking GABA degradation by GABA transaminase (GABA-T), using the suicide inhibitor *γ*-vinyl-GABA (GVG). Indeed, this drug does increase GABA levels in the brain [169, 170] and has anticonvulsant efficacy [171, 172]. Studies at the single cell level show that GVG increases miniature IPSC amplitude, consistent with a dynamic regulation of vesicular GABA concentration by the equilibrium between synthesis and degradation [173, 174]. Clinical use of GVG is, however, limited due to pathological changes of retinal cells and resulting scotoma [175].

An alternative approach suited to enhance synaptic GABA levels is the redirection of GABA uptake from glia to neurons. In glial cells, most GABA is degraded and fed into energy metabolism [176]. In contrast, neuronal GABA uptake can recycle the amino acid for use as a transmitter. It would therefore be ideal to have glia-specific GABA uptake inhibitors. Unfortunately, the molecular distinction between glial and neuronal GABA uptake is not strict, although there is some bias for GAT-1 in neurons and GAT-3 in glia [108–110].

In summary, there is no doubt that changes in GABA concentration contribute significantly to network homeostasis in health and disease. More quantitative information about sources, compartmentalization, and local concentration of GABA is urgently needed, not at least in order to develop more specific drugs for reconstituting excitation-inhibition balance in pathological situations.
